# Epidemiological insights into bovine babesiosis in South African cattle

**DOI:** 10.1007/s00436-026-08690-6

**Published:** 2026-05-07

**Authors:** Thandikhaya Bambeni, Charles Byaruhanga, Wilhelm Hein Stoltsz, Leonhard Schnittger, Raksha Vasantrai Bhoora

**Affiliations:** 1 Department of Veterinary Tropical Diseases, Faculty of Veterinary Science, University of Pretoria, Private Bag X04, Onderstepoort, 0110 South Africa; 2 Instituto de Patobiologia Veterinaria, Centre of Research in Veterinary and Agronomic Sciences, INTA-CONICET, Hurlingham, Buenos Aires 1686 Argentina; 3 National Research Council of Argentina (CONICET), Godoy Cruz 2290, CABA, (C1425FQB) República Argentina

**Keywords:** Bovine babesiosis, Epidemiology, Prevalence, Seroprevalence, TaqMan qPCR, Endemic stability

## Abstract

Bovine babesiosis, caused by *Babesia bovis* and *Babesia bigemina*, is a major cattle disease in South Africa. This study combined prospective (2022–2023) and archived (2016–2022) DNA samples to assess molecular prevalence and serological exposure to *Babesia* spp. across thirteen dip tanks in the Mnisi area (Mpumalanga), plus five additional high-risk areas: Manaleni and Boekenhouthoek (Mpumalanga), Phuthaditjaba and Harrismith (Free State), Bergville (KZN). TaqMan quantitative PCR (qPCR) and indirect fluorescent antibody testing (IFAT) were used to assess current parasitaemia and prior exposure. In Mnisi, 81/216 cattle tested positive for at least one *Babesia* spp. (38%, 95% CI: 0.31–0.44), comprising 41 single *B. bovis* infections (19%, 95% CI: 0.14–0.25), 12 single *B. bigemina* infections (6%, 95% CI: 0.03–0.09), and 28 mixed infections (13%, 95% CI: 0.09–0.18), while 135 animals were negative (63%, 95% CI: 0.56–0.69). In the remaining areas, 113/187 cattle were positive (60%, 95% CI: 0.53–0.67). The overall prevalence of *B. bigemina* was highest in the Free State (51%), while *B. bovis* was predominant in KZN (62%), with Mpumalanga showing a higher relative occurrence of *B. bovis* (32%) than *B. bigemina* (22%). Agreement analysis from Mnisi subset (*n* = 101, 2022) with paired IFAT and qPCR results revealed slight agreement for *B. bovis* (K = 0.12, 95% CI: -0.05-0.29), with McNemar’s exact test confirming systematic rather than random discordance (χ2 = 12.26, *p* = 0.00046). This supports the complementary application of both assays, as qPCR and IFAT reflect active parasitaemia and historical exposure, respectively, informing enzootic stability monitoring and control strategies.

**Supplementary Information:**

The online version contains supplementary material available at 10.1007/s00436-026-08690-6.

## Introduction

Bovine babesiosis is a vector-borne disease of significant veterinary importance, causing severe economic losses in cattle-producing regions globally (Florin-Christensen et al. 2014). The causative agents include *Babesia bovis* and *Babesia bigemina* (Schnittger et al. 2022). Both parasites are transmitted by one-host ticks of the *Rhipicephalus* genus, with *Rhipicephalus microplus* serving as the vector of both *B. bovis* and *B*. *bigemina*, while *Rhipicephalus decoloratus* transmits only *B. bigemina* (Walker et al. 2013). *Babesia bovis* is considered more pathogenic due to its ability to sequester in capillaries, resulting in more severe clinical manifestations such as anaemia, high fever, haemoglobinuria, neurological signs, and high mortality rates in cattle (Suarez et al. 2019). The presence of *R. microplus* and, consequently, *B. bovis* is influenced by various ecological and management factors, including climate change, cattle movement, and acaricide usage (Sungirai et al. 2018; Marques et al. 2020). In South Africa and remaining endemic regions, *B. bovis* infections cause substantial economic losses through decreased milk and meat productivity, reproductive inefficiencies, treatment costs and mortalities, especially in susceptible, non-immune cattle introduced into endemic areas (Mtshali and Mtshali 2013).

Molecular and serological surveys of bovine babesiosis in South Africa have shown that *B. bovis* and *B. bigemina* are present in all nine provinces, though at variable prevalences across provinces, likely reflecting the differences in the tick vector distribution and environmental factors (Terkawi et al. 2011; Mtshali and Mtshali 2013). For instance, high seroprevalence of *B. bovis* was reported in Mpumalanga and KwaZulu-Natal, while *B. bigemina* prevalence was particularly high in the Free State (87.5%) (Mtshali and Mtshali 2013). Serological assays (e.g. ELISA, IFAT) generally yielded higher estimates of infection prevalence than nested PCR in these surveys, likely because parasite densities in the blood fall below the limits of molecular detection (Terkawi et al. 2012). In chronic or subclinical infections, antibody titres may remain detectable for some time even after the parasite has been cleared, leading to seropositive but PCR-negative results. Moreover, for *B. bovis*, the sequestration of parasitised erythrocytes in the microvasculature could contribute to low circulating parasitaemia, further limiting detection by molecular assays (Terkawi et al. 2011, 2012).

The ability of nested PCR to diagnose subclinical infections is significant and has thus far contributed to determining the global distribution of *B. bovis* infections (Ganzinelli et al. 2020; Wang et al. 2020). However, nested PCR assays, while highly sensitive, are less reliable due to the increased risk of amplicon carry-over contamination. The development of quantitative real-time PCR (qPCR) has significantly improved the molecular detection and diagnosis of many organisms of veterinary importance. Detection and quantification occur in a single tube, thus eliminating the need for post-PCR manipulation and reducing the risk of contamination (Ramos et al. 2011). Real-time PCR assays developed for the diagnostic detection of *B. bovis* infections include a hydrolysis probe-based TaqMan assay targeting a conserved region of the *18S rRNA* gene (Kim et al. 2007) and a SYBR Green-based qPCR assay targeting the *msa*−2c gene, which is a highly conserved member of the merozoite surface antigen family unique to the *B. bovis* genome (Wilkowsky et al. 2003; Ramos et al. 2011). The *msa*−2c qPCR assay demonstrated higher sensitivity than conventional PCR, detecting additional positive cattle in an enzootically stable area (Ramos et al. 2011). However, in Brazil, the TaqMan-based 18S qPCR assay showed high concordance with conventional PCR across 92 field samples (Kim et al. 2007). Recently, the detection of *B. bovis* and *B. bigemina* using the TaqMan 18S qPCR assays was evaluated in Southern Africa (Stoltsz et al. 2020; Byaruhanga et al. 2023). Sequence variations between African (South Africa and Mozambique) and published *B. bovis 18S rRNA* genes were observed, particularly at the 3’-end of the *B. bovis* TaqMan qPCR forward primer (BoF) region. To enhance the diagnosis of bovine babesiosis in Southern Africa, the assay was further optimised by redesigning the *B. bovis* forward primer (BoF2) (Byaruhanga et al. 2023). The optimised assays were successfully applied to field samples from Mozambique and from the Mnisi Community area in the Mpumalanga Province, South Africa, enabling the acquisition of both occurrence data and parasitaemia estimates (Stoltsz et al. 2020; Byaruhanga et al. 2023). Improved qPCR assays, therefore, represent a crucial advancement for diagnosing bovine babesiosis and for monitoring both clinical cases and subclinical carrier animals in South African cattle populations.

The Mnisi Community, located adjacent to the Kruger National Park, is well known for its dependence on livestock production and its close interaction with diverse wildlife populations. Recent studies have highlighted the invasion of *R. microplus* and its displacement of the endemic *R. decoloratus*, a shift that may be influencing the distribution and prevalence of *Babesia* spp. within the region (Jongejan et al. 2020). In the Mnisi community, molecular surveys have reported prevalence of 44.3% for *B. bovis* and 37% for *B. bigemina* (Stoltsz et al. 2020; Byaruhanga et al. 2023). Variation in species occurrence across the area may be explained by differences in environmental conditions, particularly in the distribution and abundance of *R. microplus* across ecological zones, which, in turn, affect the transmission dynamics (Mtshali and Mtshali 2013). Building on these findings, the present study investigates the epidemiology of *Babesia* spp. in Mnisi and other high-risk areas of South Africa, aiming to provide a more comprehensive understanding of parasite distribution and inform region-specific intervention strategies.

## Materials and methods

### Sample collection

#### Sampling design and sample collection

The present study combines prospectively collected blood samples from Boekenhouthoek, Manaleni, and five dip tanks in the Mnisi community area, as well as biobanked DNA samples previously collected at various time points. The biobanked samples were selected based on availability in the freezers. Prospective sample collection followed a cross-sectional design, employing a multi-stage cluster sampling strategy for communally grazed cattle in Mpumalanga, South Africa. Dip tanks in the Mnisi community area were selected based on prior surveillance activity about the occurrence of tick species and tick-borne pathogens amongst cattle in the region, rather than through random probability sampling, with samples comprising both prospectively collected and biobanked material. Samples from the Free State and KwaZulu-Natal were drawn entirely from biobanked material, while additional Mpumalanga sites outside Mnisi were sampled prospectively. A total of 216 EDTA blood samples were collected from cattle at 13 dip tanks in the Mnisi community area, Mpumalanga Province, between 2016 and 2022 (Table 1). Additionally, 187 samples, including both biobanked DNA and freshly collected EDTA blood from cattle in the Free State, KwaZulu-Natal and other regions of Mpumalanga, were screened (Table 2). A total of 104 serum samples were collected from five of the 13 dip tanks in the Mnisi area. Blood samples were collected from cattle of mixed ages, and age-stratified sampling was not performed. As a result, calf-specific seroprevalence data required for the formal classification of enzootic stability were unavailable for this study. Because different sites were sampled at different time points across the study period, direct comparisons between localities should be interpreted with this temporal heterogeneity in mind.

**Table 1:** Bovine blood and serum samples collected from dip tanks in the Mnisi community area, Mpumalanga Province, South Africa

Dip tank	Blood samples (*n*)	Serum samples (*n*)	Collection date or period	Sample source
Utah and Dixie	14	-	November 2016	DVTD Biobank
Eglington	19	-	January 2017	DVTD Biobank
Dixie	10	-	March 2021	DVTD Biobank
Athol	10	-	January 2022	DVTD Biobank
Seville B	20	-	January 2022	DVTD Biobank
Clare A	10	-	February 2022	DVTD Biobank
Clare B	10	-	February 2022	DVTD Biobank
Shorty	10	-	March 2022	DVTD Biobank
Thlavakisa	10	-	March 2022	DVTD Biobank
Welverdiend A	20	20	November 2022	This study
Welverdiend B	22	22	November 2022	This study
Athol	21	21	November 2022	This study
Seville A	20	20	November 2022	This study
Ludlow A	20	21	November 2022	This study
Total	**216**	**104**

**Table 2:** Bovine blood samples collected from selected locations in Free State, KwaZulu-Natal, and Mpumalanga provinces, South Africa

Province	Locality	Samples (*n*)	Collection period	Sample source
Free State	Harrismith	50	October - November 2021	DVTD Biobank
	Phuthaditjhaba	50	October - November 2021	DVTD Biobank
KwaZulu-Natal	Bergville	50	October - November 2021	DVTD Biobank
Mpumalanga	Boekenhouthoek	17	November 2023	This study
	Manaleni	20	November 2023	This study
Total		**187**		

### Sample processing

All samples collected from the Mnisi community area were processed at the Hans Hoheisen Wildlife Research Station (HHWRS). Retrospective EDTA samples were processed at the Research and Training Laboratories of the Department of Veterinary Tropical Diseases. Bovine serum samples were centrifuged at 3000 rpm for 10 min at 4 °C to separate the serum from the red blood cells. The serum was then aliquoted into sterile 1.5 ml microcentrifuge tubes and heat-inactivated at 60 °C for 30 min to inactivate any potential pathogens. After heat inactivation, the serum samples were stored at −20 °C.

#### DNA extraction

Genomic DNA was isolated from 200 µl of blood using the PureLink™ Genomic DNA Mini Kit (ThermoFisher Scientific™, Johannesburg, South Africa) according to the manufacturer’s guidelines. The extracted genomic DNA and inactivated bovine sera were transported to the Department of Veterinary Tropical Diseases Research and Training (DVTD-R&T) laboratories with the necessary permits and stored at − 20 °C for further analysis.

### Indirect fluorescent antibody test for *Babesia bovis*

The Indirect Fluorescent Antibody Test (IFAT) was carried out at the Agricultural Research Council’s Onderstepoort Veterinary Research facility (Onderstepoort, South Africa) using an in-house protocol. *Babesia bovis*-infected red blood cells (RBCs) were used as antigens to coat the IFAT slides. Serum samples were screened at 2-fold serial dilutions starting at 1:40. A titer of 1:80 or higher was considered seropositive. Bound antibodies were detected using fluorescein isothiocyanate (FITC)-conjugated sheep anti-bovine IgG antibodies, and the samples were examined with a fluorescent microscope.

### Detection of *B. bovis* and *B. bigemina* using TaqMan qPCR assays

The samples were screened for bovine babesiosis using published TaqMan quantitative real-time PCR (qPCR) assays targeting the *18 S rRNA* genes of *B. bovis* and *B. bigemina* (Kim et al. 2007; Byaruhanga et al. 2023). The assay was adapted for use on the QuantStudio™ 5 Real-Time PCR system (ThermoFisher Scientific™, South Africa) with minor modifications. The two assays were run independently, each containing 10 µl KAPA probe Fast Universal Master Mix (KAPA Biosystems, Cape Town, South Africa), 0.5 µM of each oligonucleotide primer and 0.25 µM of the FAM –(*B. bovis*) or VIC– (*B. bigemina*) TaqMan probe (Table 3). The positive controls included DNA isolated from South African *B. bovis* and *B. bigemina* vaccine strains (Onderstepoort Biological Products, South Africa), while double-distilled water served as the negative control. The cycling conditions included an initial denaturation of 20 s at 95 °C, followed by 45 two-step cycles of 1 s at 95 °C and a final extension of 60 s at 57 °C (Kim et al. 2007).

**Table 3:** Primers and probes for qPCR detection of *Babesia bovis* and *B. bigemina*

Species	Primer/Probe designation	Sequence (5’ → 3’)	Reference
*B. bovis*	BoP	6-FAM-CCTTGTATGACCCTGTCGTACCGTTGG-TAMRA	(Kim et al. 2007)
	BoF2	GGTTTCGCCTGTATAATTG	(Byaruhanga et al. 2023)
	BoR	AGTCGTGCGTCATCGACAAA	(Kim et al. 2007)
*B. bigemina*	BiP	VIC-TTGGAATGATGGTGATGTACAACCTCA-TAMRA	
	BiF	AATAACAATACAGGGCTTTCGTCT	
	BiR	AACGCGAGGCTGAAATACAACT	

### Statistical analysis

Statistical analyses were performed either using the R statistical software version 4.5.1, with the packages “lmtest” and “psych” at a significance level of 5% (R Core Team 2025) or IBM SPSS Statistics (Version 31). The 95% confidence intervals for the proportion of *B. bovis*, *B. bigemina* and mixed infections were calculated using the Wilson score method in Epitools (Brown et al. 2001). Fisher’s exact test was used to assess the significance of the association between *B. bovis* and *B. bigemina* infections and the occurrence of mixed infections at each sampling site using IBM SPSS Statistics (Version 31). Agreement between *B. bovis* detection by qPCR and IFAT was assessed using Cohen’s kappa statistic, while the McNemar’s exact test with a continuity correction was applied to evaluate whether disagreement between the two tests (qPCR, IFAT) in the detection of *B. bovis* was statistically significant. In addition, a Generalised Linear Model and likelihood ratio tests were used to evaluate the association between *B. bovis* exposure (as indicated by IFAT results) or infection status (determined by qPCR for *B. bovis* and *B. bigemina*) and the location of origin (dip tank).

## Results

### Molecular detection and distribution of *B. bovis* and *B. bigemina*

#### Prevalence and infection types in the Mnisi community area

A total of 216 blood samples collected from cattle across 13 dip tanks between 2016 and 2022 were screened for *Babesia* spp. using the TaqMan 18 S qPCR assay. Overall, 81 animals (38%, 95% CI: 0.31–0.44) tested positive, including 41 (19%, 95% CI: 0.14–0.25) with single *B. bovis* infections, 12 (6%, 95% CI: 0.03–0.09) with single *B. bigemina* infections, and 28 (13%, 95% CI: 0.09–0.18) with mixed infections. The remaining 135 animals (63%, 95% CI: 0.56–0.69) tested negative.

#### Spatial variation across Mnisi dip tanks

**Fig. 1:**
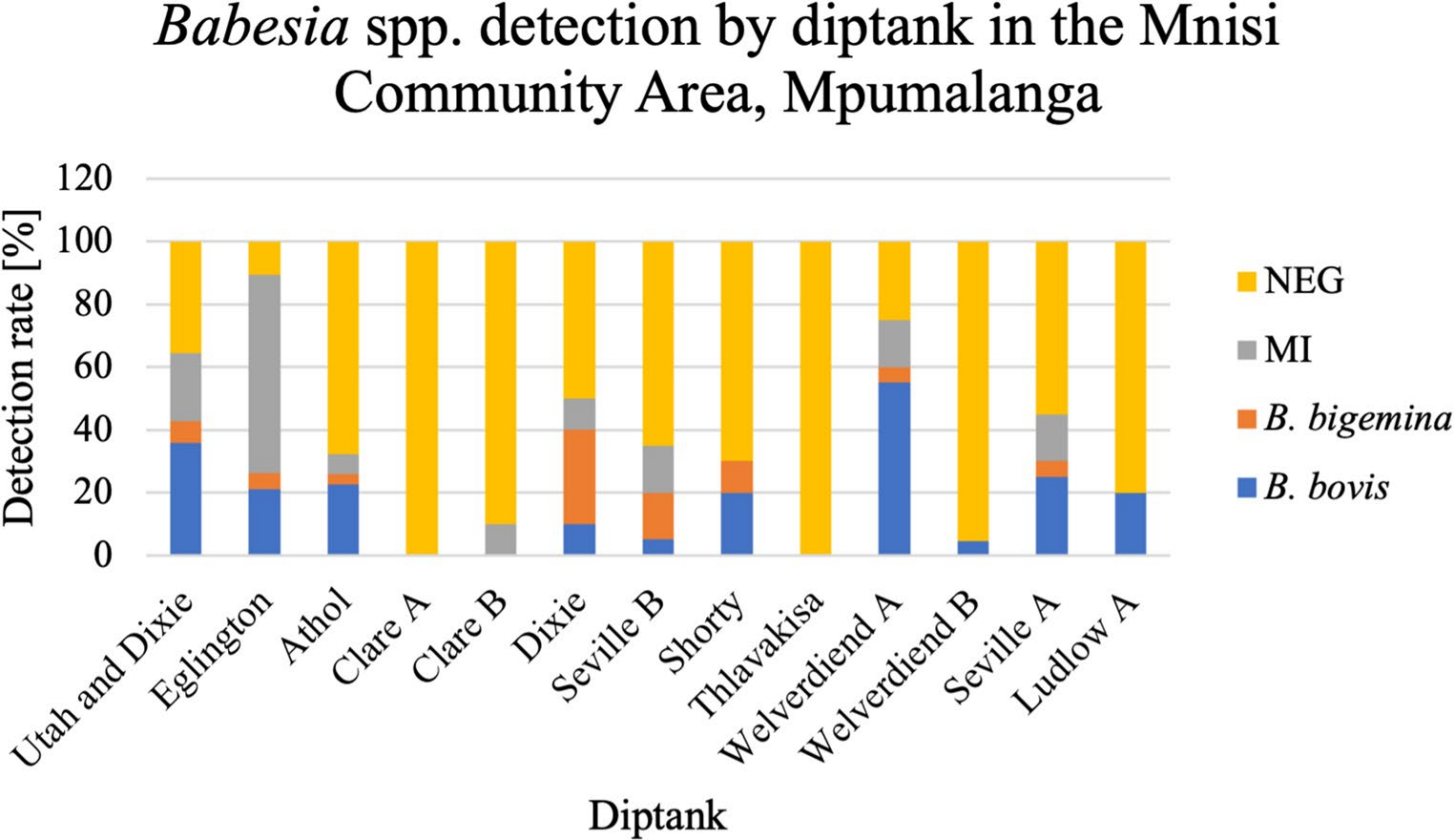
Stacked bar graph showing the distribution of *B. bovis (blue)*, *B. bigemina (orange)*, mixed infections (MI, *grey*), and negative animals (NEG, *yellow*) across selected dip tanks within the Mnisi community area, Mpumalanga Province, South Africa. Sites are sorted by the year of sample collection (2016–2022) to illustrate temporal trends in prevalence

Considerable variation was observed across dip tanks (Fig. 1). The highest combined prevalence of *Babesia* spp. was recorded in Eglinton (17/19, 90%, 95% CI: 0.69–0.97), followed by Welverdiend A (15/20, 75%, 95% CI: 0.53–0.89), Utah and Dixie (9/14, 64%, 95% CI: 0.39–0.84), while Clare A and Thlavakisa had no *Babesia*-positive cases.

Mixed infections were most frequent in Eglinton (63%, 95% CI: 0.41–0.81), followed by Utah & Dixie (21%, 95% CI: 0.08–0.48) and then Seville A, Seville B and Welverdiend A (15%, 95% CI: 0.05–0.36) (Fig. 1). Notably, single *B. bovis* detection was the highest in Welverdiend A (55%, 95% CI: 0.34–0.74), followed by Utah and Dixie (36%, 95% CI: 0.16–0.61), while single *B. bigemina* was high in Dixie (30%, 95% CI: 0.11–0.60). Overall, single *B. bovis* was more frequently detected than single *B. bigemina* infections across all sites.

#### Temporal trends in Mnisi sampling

Samples from Eglinton and Utah & Dixie were collected between 2016 and 2017, whereas Athol was sampled in 2021–2022, and all other sites were sampled in 2022 (Fig. 1). This distribution provides a temporal snapshot of infection prevalence across the Mnisi community area.

#### Statistical associations in Mnisi cattle

Generalised linear models indicated a statistically significant effect of dip tank location on the overall distribution of *Babesia* pathogens in cattle from the Mnisi area (Supplementary data, Table S1); however, the relatively small sample sizes per site and non-probability (convenience) sampling of biobanked DNA samples, which were selected based on their availability, limit the strength of this conclusion.

The co-occurrence of *B. bovis* and *B. bigemina* infections was evaluated at two levels: across the Mnisi area and within individual dip tanks. The overall analysis investigates whether the two infections tend to co-occur within the population, while the site-specific analysis examines whether co-occurrence at each dip tank exceeds what would be expected by chance based on local infection rates. First, an overall chi-square test was performed on pooled data from all Mnisi dip tanks. This test confirmed a significant association between the two infections (χ^2^ = 30.59, *p* < 0.0001) (Table 4), indicating that the occurrence of one species was not independent of the other when data were combined across sites. However, site-level analysis using Fisher’s exact test showed no significant deviation from independence at any individual dip tank (*p* > 0.05; Supplementary data, Table S3). This apparent discrepancy reflects that at the population level, cattle infected with one species are more likely to also carry the other, consistent with shared exposure to tick vectors across Mnisi as a whole. At the dip tank level, sample sizes are small and infection pressures vary, so the co-occurrence signal becomes statistically undetectable within individual sites. At Seville B, the calculated conditional probability of co-infections between *B. bovis* and *B. bigemina* did not reach statistical significance (*p* = 0.06), although mixed infections occurred more frequently than expected under independence. This borderline result likely arises from a small sample size, which reduces statistical power rather than suggesting a true absence of association. The observed effect, with mixed infections exceeding expected frequencies, is consistent with the pattern observed at other high-prevalence sites in Mnisi. Statistical testing was not performed for Clare A, Thlavakisa and Ludlow A, as infection status was constant within these sites, preventing assessment of independence.

**Table 4:** Proportions of *Babesia bovis* and *Babesia bigemina* infections amongst cattle sampled at 13 dip tanks

		*Babesia bigemina* (no. and % of positive)		
		Positive	Negative	**Total**
***Babesia bovis*** **(no. and % of positive)**	Positive	28 (13)	41 (19)	**69 (32)**
	Negative	12 (6)	135 (63)	**147 (68)**
	**Total**	**40 (19)**	**176 (82)**	**216**

Infections were determined using two species-specific quantitative real-time PCR assays. The association between *B. bovis* and *B. bigemina* infections was assessed using a chi-square test of independence (χ^2^ = 30.59, df = 1, *p* < 0.0001)

#### Proportions of single and mixed infections in five localities across three provinces of South Africa

**Fig. 2:**
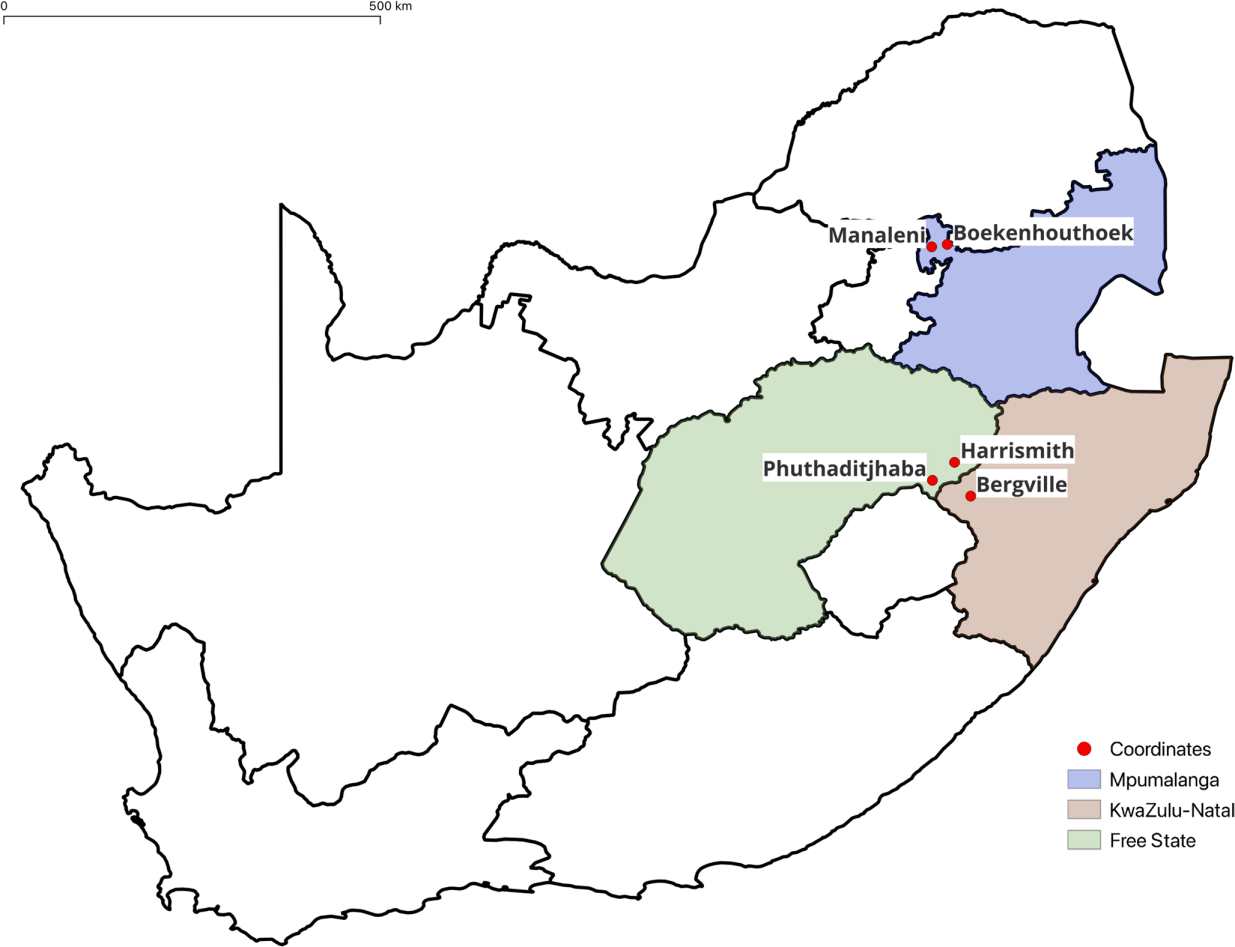
Map of South Africa illustrating the five locations in three provinces where samples were collected. The map was created using QGIS version 3.40.5-Bratislava from Simple maps under the Creative Commons Attribution 4.0 license

To assess the broader distribution of *Babesia* spp., an additional 187 samples collected from five localities outside the Mnisi area, including Phuthaditjaba and Harrismith (Free State), Bergville (KZN), and Manaleni and Boekenhouthoek (Mpumalanga), were screened (Fig. 2).

**Fig. 3:**
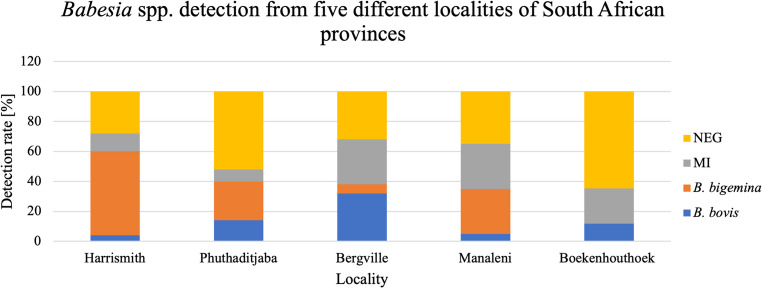
Clustered bar chart showing the percentages of cattle samples positive for *B. bovis* (% *B. bovis*, *blue*), *B. bigemina* (% *B. bigemina*, *orange*), co-infections of *B. bovis* and *B. bigemina* (% Mixed, *grey*), as well as negative samples (NEG, *yellow*) across five different localities

Of the 187 cattle tested, 113 (60%, 95% CI: 0.53–0.67) were positive for at least one *Babesia* spp. The highest proportion of single *B. bovis* infections occurred in Bergville (32%, 95% CI: 0.21–0.46), followed by Phuthaditjaba (14%, 95% CI: 0.07–0.26), Boekenhouthoek (12%, 95% CI: 0.03–0.34), Manaleni (5%, 95% CI: 0.01–0.24), and Harrismith (4%, 95% CI: 0.01–0.13) (Fig. 3). In contrast, single *B. bigemina* infections were most frequent in Harrismith (56%, 95% CI: 0.42–0.69), followed by Manaleni (30%, 95% CI: 0.15–0.52), Phuthaditjaba (26%, 95% CI: 0.16–0.40), Bergiville (6%, 95% CI: 0.02–0.16) and absent in Boekenhouthoek (Fig. 3). Mixed infections were highest in Bergville (30%, 95% CI: 0.19–0.44), and Manaleni (30%, 95% CI: 0.15–0.52), followed by Boekenhouthoek (24%, 95% CI: 0.10–0.47), Harrismith (12%, 95% CI: 0.06–0.24), and Phuthaditjaba (8%, 95% CI: 0.03–0.19) (Fig. 3). These findings suggest regional variation in transmission dynamics and vector distribution.

#### Concordance analysis of *B. bovis* and *B. bigemina* infections across five localities

The co-occurrence of *B. bovis* and *B. bigemina* infections within individual cattle across five localities in three provinces was assessed using a chi-square test (Table 5). No statistically significant association between the two pathogens was detected overall across the five sites (χ^2^ = 3.32, *p* = 0.068). The absence of an overall association does not indicate that co-occurrence is uniformly absent across sites, but rather that the pattern is heterogeneous, with co-occurrence concentrated at specific localities. Location-specific analysis further revealed variability in the co-occurrence of *B. bovis* and *B. bigemina* infections (Supplementary data, Table S3). No significant association was detected at Phuthaditjaba, Manaleni or Harrismith (*p* > 0.05), where observed frequencies of mixed infections closely matched expected values. However, significant deviations from independence were observed at Bergville (*p* = 0.03) and Boekenhouthoek (*p* = 0.01), with mixed infections occurring more frequently than expected, suggesting localised conditions that may favour co-transmission of both species at these sites.

**Table 5:** Proportions of *B. bovis* and *B. bigemina* infections in cattle across five localities in three provinces of South Africa

		*Babesia bigemina* (no. and % of positive)		
		Positive	Negative	**Total**
***Babesia bovis*** **(no. and % of positive)**	Positive	35 (18.7)	28 (14.9)	**63 (33.7)**
	Negative	50 (26.7)	74 (39.6)	**124 (66.3)**
	**Total**	**85 (45.5)**	**102 (54.5)**	**187**

Infections were determined using two species-specific quantitative real-time PCR assays. The association between *B. bovis* and *B. bigemina* infections was assessed using a chi-square test of independence (χ^2^ = 3.32, df = 1, *p* = 0.068)

### Detection of *B. bovis* antibodies using IFAT

IFAT performed on a subset of cattle (*n* = 101) from five dip tanks showed a similar trend of seroprevalence across four sites (Ludlow A: 50%, Athol: 57%, Seville A: 55%, Welverdiend B: 50%; Table 6). The estimates at Welverdiend A (80%) were substantially higher than those at other sites but not statistically significant (95% CI: 0.98–16.27, *p* = 0.053), with a wide confidence interval that includes values less than 1, consistent with the relatively limited sample sizes (Table 6). Overall, dip tank location was not significantly associated with *B. bovis* seropositivity (Likelihood ratio test, *p* = 0.243).

**Table 6:** IFAT results for *Babesia bovis* seroprevalence in cattle sampled from five dip tanks, showing the number of positive and negative results, total animals tested, and the calculated seroprevalence

Dip tank	Samples tested (*n*)	No. and percentage of positive samples	Odds ratio and 95% CI	Wald *p*-value	Likelihood ratio *p*-value
Ludlow A	20	10 (50)	Reference		
Athol	21	12 (57)	1.33 (0.39–4.57)	0.647	
Seville A	20	11 (55)	1.22 (0.35–4.24)	0.752	
Welverdiend A	20	16 (80)	4.00 (0.98–16.27)	0.053	
Welverdiend B	20	10 (50)	1.00 (0.29–3.45)	1.000	0.243

Comparison of qPCR and IFAT results for *B. bovis* by Cohen’s Kappa Statistic showed a slight agreement (K = 0.12, 95% CI: −0.05-0.29). The McNemar’s exact test was also conducted and showed that the disagreement between the two tests (qPCR and IFAT) in detecting *B. bovis* was statistically significant (χ2 = 12.26, *p* = 0.00046), and therefore not due to chance. The 101 cattle samples were tested by both IFAT and qPCR, and 23 of 101 (22.8%) were positive by both methods, 36 of 101 (35.6%) were IFAT+/qPCR-, and 11 of 101 (10.9%) were IFAT-/qPCR+ (Table 7). These results indicate widespread exposure in the area, suggesting a mixture of past and ongoing infections, rather than a perfect alignment between the two diagnostic methods.

**Table 7:** A 2 × 2 contingency table comparing qPCR and IFAT results for the detection of *B*. *bovis* DNA and antibodies, respectively, in bovine blood samples; agreement was assessed using Cohen’s kappa

	qPCR Positive (%)	qPCR Negative (%)	Total
IFAT Positive (%)	23 (22.8)	36 (35.6)	59
IFAT Negative (%)	11 (10.9)	31 (30.7)	42
Total	34	67	101

Cohen’s Kappa (K = 0.12, 95% CI: −0.05-0.29)

## Discussion

Although the non-probability (convenience) sampling design and temporal heterogeneity across sites limit the generalisability of the findings, this study provides a molecular and serological assessment of *B. bovis* and *B. bigemina* infections in cattle across the Mnisi Community Area and five additional localities in South Africa. By combining TaqMan qPCR and IFAT, the data reveal substantial spatial variation in prevalence, evidence of long-term exposure in many herds, and possible shifts in vector-pathogen ecology that have implications for transmission intensity and herd immunity across parts of the country.

Bovine babesiosis has long been recognised as a major constraint to cattle production in South Africa. Early reviews emphasised the disease’s economic importance and the central role of tick vectors in its control. These foundational observations emphasise the importance of monitoring both infection and exposure for livestock health and trade (De Vos 1979).

Enzootic stability describes a state in which the inoculation rate of *Babesia* spp. by the tick vector is sufficient to immunise a majority of susceptible calves before the loss of maternal immunity (Mahoney and Ross 1972; De Vos 1979). Operationally, stability has been defined as seropositivity exceeding 75% in calves aged between nine and twelve months, reflecting high transmission rates during the protected window of age-related resistance (Smith et al. 2000). Enzootic instability, in contrast, arises when transmission rates are insufficient to expose the entire cohort, leaving a portion of the herd susceptible to clinical disease after that protective window closes (De Vos 1979). Formal classification of sites as stable or unstable requires age-stratified seroprevalence data and, ideally, tick infestation indices; neither of these was available in the present cross-sectional dataset. The present study, therefore, does not formally classify sites according to these criteria. Similar to Rikhotso et al. (2005), who used serological data from mixed-age communal cattle in the Bushbuckridge region of Limpopo Province to evaluate endemic stability under different dipping strategies, the present study interprets seroprevalence and prevalence patterns as broadly indicative of transmission intensity rather than as formal classifications based on age-stratified calf data. However, unlike Rikhotso et al. (2005), the cross-sectional design and temporal variations across sampling sites in this study prevent any directional comparisons of seroprevalence changes over time.

The highest prevalence of *B. bigemina* was recorded in the Free State (51.0%), followed by KZN (36.0%) and Mpumalanga (22.1%). The predominance of *B. bigemina* in the Free State is consistent with the wide distribution of *R. decoloratus*, the main vector of this parasite, as reported in previous studies (Dreyer et al. 1998; Mtshali et al. 2004; Mtshali and Mtshali 2013). However, the observed prevalence values were significantly lower than those previously reported by a nested PCR assay, which showed infection rates of 87.5% in the Free State and 84.6% in KZN (Mtshali and Mtshali 2013). Historically, high levels have been interpreted as evidence of enzootic stability, where at least 81% of animals are exposed, providing a degree of herd immunity and reducing the incidence of clinical disease (Mtshali and Mtshali 2013).

The lower prevalence observed in this study may suggest a shift away from conditions previously associated with enzootic stability for *B. bigemina*, potentially increasing the risk of clinical babesiosis, particularly in young or immunologically naïve animals. Factors proposed in literature as possible contributors to such a change include intensified tick control measures that reduce *R. decoloratus* populations, ecological displacement of *R. decoloratus* by *R. microplus*, changes in grazing patterns or cattle management practices, and reduced early-life exposure leading to lower herd immunity (Springer et al. 2020; Slayi and Mpisana 2025). These remain hypotheses drawn from published literature rather than findings of the present study, which did not estimate tick burden or collect climate data to directly test these mechanisms. Further investigation is needed to determine whether the observed prevalence reflects a genuine epidemiological shift or is affected by seasonal, management, or sampling factors.

For *B. bovis*, the highest prevalence occurred in KZN (62.0%), followed by Mpumalanga (32.4%) and the Free State (19.0%). The high prevalence of *B. bovis* in KZN correlates with the widespread distribution of *R. microplus*, the invasive and highly efficient vector of this parasite, which thrives in humid coastal and subtropical environments (Mtshali and Mtshali 2013; Nyangiwe et al. 2017; Makwarela et al. 2024). These findings are consistent with reports that *R. microplus* remains well-established in coastal provinces and is gradually spreading inland into parts of Mpumalanga and the Free State (Madder et al. 2011; Nyangiwe et al. 2017).

In Mnisi, *B. bovis* prevalence was broadly comparable to previous findings reporting a 44.3% prevalence using an optimised quantitative PCR assay (Byaruhanga et al. 2023), suggesting ongoing transmission sustained by the overlapping presence of *R. microplus* and *R. decoloratus* (Jongejan et al. 2020). Interestingly, unlike previous studies that reported higher *B. bigemina* and lower *B. bovis* prevalence in Mpumalanga, our data reveal the opposite trend. This reversal may reflect a possible shift in vector dominance, potentially driven by the displacement of *R. decoloratus* by the more invasive *R. microplus* (Tønnesen et al. 2004). Such displacement could alter the transmission dynamics and the relative prevalence of *Babesia* species, especially in regions where these tick species overlap. However, the present study did not collect new tick or climate data to directly test this mechanism, and these explanations should be regarded as hypotheses informed by prior literature rather than conclusions of this study. Additionally, the sampling period of the present study may have differed from that of previous studies reporting higher prevalence, which could partly explain discrepancies in infection rates. The lower *B. bigemina* prevalence in the current Mpumalanga samples may indicate reduced *R. decoloratus* activity, while continuous detection of *B. bovis* suggests that *R. microplus* may be responsible for ongoing transmission, as it can maintain higher infestation levels throughout the year in favourable climatic conditions.

An important consideration is whether these results are consistent with the conditions for enzootic stability. In Mnisi and other localities with high prevalence and seropositivity, such as Bergville and Manaleni, mixed infections reached 30%, and the patterns are broadly consistent with ongoing transmission pressure associated with stable endemicity, where continuous exposure leads to broad herd immunity. Bearing in mind that these sites were not sampled simultaneously and that selection was based on prior surveillance activity, direct epidemiological comparisons between localities carry some uncertainty. However, this was not consistent across all sites. In Harrismith, *B. bigemina* predominated, largely as single infections, while overall prevalence was lower than at other sites, suggesting reduced infection pressure. Conversely, in Clare A and Thlavakisa (Mnisi), where no infections were detected, cattle remain susceptible to clinical disease outbreaks, reflecting conditions more consistent with enzootic instability. Thus, while some herds may benefit from conditions broadly associated with enzootic stability, others face conditions suggestive of instability and heightened clinical risk.

The site-specific co-occurrence patterns support this interpretation. At Bergville and Boekenhouthoek, mixed infections occurred significantly more frequently than expected under independence (*p* = 0.03 and *p* = 0.01, respectively), suggesting localised conditions favouring simultaneous transmission of both species, likely reflecting higher tick vector diversity or burden at these sites. At Harrismith, Phuthaditjaba, and Manaleni, where infections appeared independent, the predominance of single-species infections, particularly *B. bigemina* at Harrismith, suggests that transmission dynamics may be driven by a single dominant vector species rather than overlapping tick populations. At Seville B in Mnisi, mixed infections exceeded expected frequencies despite the borderline p-value (*p* = 0.06), which most plausibly reflects insufficient sample size rather than a true biological absence of co-occurrence. The direction of this effect is consistent with the pattern observed at higher-prevalence sites in Mnisi and warrants further investigation with a larger sample from this dip tab.

Previous studies from Argentina and Indonesia have reported that mixed *B. bovis* and *B. bigemina* infections generally occur at frequencies consistent with expectations under independence (Guswanto et al. 2017; Ganzinelli et al. 2020). In agreement with these findings, the present study shows that across most sampling locations in South Africa, observed frequencies of mixed infections did not differ significantly from expected values, indicating that *B. bovis* and *B. bigemina* infections occur independently (Supplementary data, Table S3). Importantly, significant co-infections were restricted to a small number of sites, highlighting geographical variation rather than consistent epidemiological dependence between the two parasites. Such localised deviations are likely driven by location-specific ecological and management factors, including differences in vector composition, transmission intensity and host exposure. Given the distinct vector competence and distributions of *R. microplus* and *R. decoloratus*, variations in their relative occurrence may provide opportunities for co-transmission without implying direct biological interaction between the pathogens. Collectively, these findings support the conclusion that mixed *Babesia* infections arise from shared exposure to competent vectors, with local ecological factors determining location-specific patterns of co-infections.

Our overall *B. bovis* prevalence of 35.1%, derived from only three provinces in South Africa, is approximately similar to the 35.5% prevalence reported in a previous study that included samples from all nine provinces (Mtshali and Mtshali 2013; Mtshali et al. 2014). However, prevalence estimates vary widely depending on the diagnostic methods used, study location, and herd type examined. Our IFAT results (58.4%) fall within the range previously reported for South African cattle, where provincial surveys have identified moderate to high seroprevalence for *B. bovis* (Terkawi et al. 2011). A serological survey reported antibody prevalence ranging from 15% to over 70%, particularly in communal grazing systems where tick challenge is higher (Terkawi et al. 2011). In contrast, some commercial farms in the Eastern Cape showed very low detection rates (1.9%) for *B. bovis* (Marais 2020), likely due to strict tick control and predominance of *R. decoloratus*.

When comparing molecular and serological data from Mnisi, patterns emerge that are consistent with ongoing transmission and partial population-level immunity. At dip tanks such as Athol, Welverdiend A, and Seville A, qPCR prevalence (33–70%) closely mirrored seroprevalence (55–80%), suggesting active circulation and the maintenance of herd immunity. In contrast, at sites such as Welverdiend B and Ludlow A, low qPCR positivity (≤ 20%) alongside moderate seropositivity (50%) indicated historical exposure without substantial current transmission. In addition to the slight agreement between qPCR and IFAT results (K = 0.12, 95% CI: −0.05-0.29), McNemar’s exact test confirmed that the discordance between the two methods was statistically significant (χ2 = 12.26, *p* = 0.00046), confirming that the difference in results is not due to chance alone (Romero-Salas et al. 2016). The overall discordance between qPCR and IFAT results is consistent with the expected biology and prior reports (Romero-Salas et al. 2016; Ganzinelli et al. 2020), where IFAT measures historical exposure while qPCR detects current parasitaemia and is often more sensitive for acute or low-level infections. This significant disagreement is biologically plausible, as qPCR detects circulating *B. bovis* DNA and therefore reflects active parasitaemia (Byaruhanga et al. 2023), while IFAT detects antibodies generated by the host following pathogen exposure (Terkawi et al. 2011; Ganzinelli et al. 2020). Antibodies can be present due to previous exposure in the absence of active infection or due to current infection. Even in active infections, antibody levels depend on the duration of infection, with recent infections less likely to produce detectable immune responses. On the other hand, antibodies can persist in the blood long after the pathogen has been eliminated or even cleared, meaning that the parasitaemia may fall below the detection threshold for qPCR (Calder et al. 1996; Terkawi et al. 2011, 2012). These findings highlight the importance of integrating molecular and serological approaches for assessing infection dynamics, particularly in heterogeneous endemic settings.

These findings suggest that the epidemiology of bovine babesiosis in South Africa is highly localised, influenced by ecological variations in tick vector distribution, host management practices, and environmental factors. Enzootic stability may operate in high-prevalence areas but appears fragile or absent in others. This variety highlights the importance of implementing surveillance and control strategies tailored to specific regions. In areas with patterns consistent with stable endemicity, interventions should aim to maintain the equilibrium by carefully balancing tick control with vaccination. In areas with patterns suggestive of instability, more proactive protection of naïve herds is essential. These interpretations are further constrained by the non-probabilistic sampling approach, temporal heterogeneity in sampling and the small sample sizes at some localities, particularly for serological data. Larger, longitudinal studies across all nine provinces are required to confirm whether the observed patterns represent true epidemiological shifts or are influenced by temporal, management, or sampling factors.

## Conclusion

In conclusion, this study demonstrates substantial spatial heterogeneity in *B. bovis* and *B. bigemina* infections across three South African provinces, with prevalence and seropositivity patterns suggesting that conditions for enzootic stability are not uniformly maintained. The significant discordance between qPCR and IFAT highlights the value of combining the assays to monitor transmission dynamics in endemic settings. Region-specific surveillance and control strategies are needed, and the drivers of observed epidemiological variation, including possible vector displacement, warrant further investigation through targeted studies collecting tick burden and environmental data.

## Supplementary Information

Below is the link to the electronic supplementary material.

Supplementary File 1 (DOCX 41.7 KB)

## Data Availability

The data supporting the findings of this study are included within the article and its supplementary materials.
