# Supplementary material for: Epidemiological insights into bovine babesiosis in South African cattle
Source: Parasitol Res. 2026 May 7;125(1):68. doi: 10.1007/s00436-026-08690-6 (PMC13246924; doi:10.1007/s00436-026-08690-6)
Supplement: Supplementary file 1 — (DOCX 41.7 KB) [file 436_2026_8690_MOESM1_ESM.docx]

**Table S1**: qPCR prevalence of *Babesia* infections in cattle by sampling location (dip tank)

| **Location** | **No. of cattle samples (N)** | **Infection type** | **Positive N (%)** | **Odds ratio (95% CI)** | ***p*-value** |
| --- | --- | --- | --- | --- | --- |
| Clare B | 10 | *B. bovis*  *B. bigemina*  Mixed | 1(10.0)  1(10.0)  1(10.0) | Reference |  |
| Welverdiend B | 22 | *B. bovis*  *B. bigemina*  Mixed | 1(4.5)  0(0.0)  0(0.0) | 0.43(0.02-7.63)  <0.01(0-Inf)  <0.01(0-Inf) | 0.56  0.99  0.99 |
| Utah A & Dixie | 14 | *B. bovis*  *B. bigemina*  Mixed | 8(57.1)  4(28.6)  3(21.4) | 12.0(1.18-122.27)  3.6(0.34-38.48)  2.45(0.22-27.84) | 0.04  0.29  0.47 |
| Athol | 31 | *B. bovis*  *B. bigemina*  Mixed | 9(29.0)  3(9.7)  2(6.5) | 3.68(0.41-33.45)  0.96(0.09-10.47)  0.62(0.05-7.67) | 0.25  0.98  0.71 |
| Clare A | 10 | *B. bovis*  *B. bigemina*  Mixed | 0(0.0)  0(0.0)  0(0.0) | <0.01(0-Inf)  <0.01(0-Inf)  <0.01(0-Inf) | 0.99  0.99  0.99 |
| Dixie | 10 | *B. bovis*  *B. bigemina*  Mixed | 2(20.0)  4(40.0)  1(10.0) | 2.25(0.17-29.77)  6.0(0.53-67.65)  1.0(0.05-18.57) | 0.54  0.15  1.00 |
| Eglinton | 19 | *B. bovis*  *B. bigemina*  Mixed | 16(84.2)  13(68.4)  12(63.2) | 48.0(4.33-532.29)  19.5(1.99-190.88)  15.43(1.59-148.82) | 0.002  0.01  0.02 |
| Ludlow A | 20 | *B. bovis*  *B. bigemina*  Mixed | 4(20.0)  0(0.0)  0(0.0) | 2.25(0.22-23.32)  <0.01(0.0-Inf)  <0.01(0-Inf) | 0.49  0.99  0.99 |
| Seville A | 20 | *B. bovis*  *B. bigemina*  Mixed | 8(40.0)  4(20.0)  3(15.0) | 6.0(0.63-57.0)  2.25(0.22-23.32)  1.59(0.14-17.56) | 0.12  0.49  0.71 |
| Seville B | 20 | *B. bovis*  *B. bigemina*  Mixed | 4(20.0)  6(30.0)  3(15.0) | 2.25(0.22-23.32)  3.86(0.39-37.58)  1.59(0.14-17.56) | 0.49  0.25  0.71 |
| Shorty | 10 | *B. bovis*  *B. bigemina*  Mixed | 2(20.0)  1(10.0)  0(0.0) | 2.25(0.17-29.77)  1.00(0.05-18.57)  <0.01(0-Inf) | 0.54  1.00  0.99 |
| Thlavakisa | 10 | *B. bovis*  *B. bigemina*  Mixed | 0(0.0)  0(0.0)  0(0.0) | <0.01(0-Inf)  <0.01(0-Inf)  <0.01(0-Inf) | 0.99  0.99  0.99 |
| Welverdiend A | 20 | *B. bovis*  *B. bigemina*  Mixed | 14(70.0)  4(20.0)  3(15.0) | 21.0(2.15-204.61)  2.25(0.22-23.32)  1.59(0.14-17.56) | 0.009  0.49  0.71 |

**Table S2**: Proportions and odds ratios for qPCR-detected *Babesia* infections in cattle by locality and province

| **Province** | **Locality** | **No. of cattle samples (N)** | **Infection type** | **Positive N (%)** | **Odds ratio (95% CI)** | ***p*-value** |
| --- | --- | --- | --- | --- | --- | --- |
| Free State | Harrismith | 50 | *B. bovis*  *B. bigemina*  Mixed | 8(16.0)  34(68.0)  6(12.0) | Reference |  |
|  | Phuthaditjaba | 50 | *B. bovis*  *B. bigemina*  Mixed | 11(22.0)  17(34.0)  4(8.0) | 1.48(0.54-4.06)  0.24(0.11-0.56)  0.64(0.17-2.41) | 0.45  0.009  0.51 |
| Mpumalanga | Boekenhouthoek | 17 | *B. bovis*  *B. bigemina*  Mixed | 6(35.3)  4(23.5)  4(23.5) | 2.86(0.82-9.99)  0.14(0.04-0.51)  2.26(0.55-9.23) | 0.09  0.003  0.26 |
|  | Manaleni | 20 | *B. bovis*  *B. bigemina*  Mixed | 7(35.0)  12(60.0)  6(30.0) | 2.83(0.86-9.29)  0.71(0.24-2.07)  3.14(0.87-11.32) | 0.09  0.525  3.14 |
| KwaZulu-Natal | Bergville | 50 | *B. bovis*  *B. bigemina*  Mixed | 31(62.0)  18(36.0)  15(30.0) | 8.57(3.32-22.09)  0.26(0.12-0.61)  3.14(1.10-8.94) | <0.01  0.002  0.03 |

**Table S3:** Observed and expected frequencies of *B. bovis*-*B. bigemina* co-infection and tests of independence by site

| No | Locations | No. of sample | Positive samples (N) | | | Observed (N) | | | | Expected (N) | | | | *Fisher’s Exact test* (*p*-value) |
| --- | --- | --- | --- | --- | --- | --- | --- | --- | --- | --- | --- | --- | --- | --- |
|  |  |  | *B. bovis* | *B. bigemina* | Mixed | *bov + big +* | *bov + big -* | *bov - big +* | *bov - big -* | *bov + big +* | *bov + big -* | *bov - big +* | *bov - big -* |  |
|  |  |  | *qPCR* | *qPCR* | *qPCR* |  |  |  |  |  |  |  |  |  |
| 1 | Utah and Dixie | 14 | 5 | 1 | 3 | 3 | 5 | 1 | 5 | 2,30 | 5,70 | 1,70 | 4,30 | 0,58 |
| 2 | Eglington | 19 | 4 | 1 | 12 | 12 | 4 | 1 | 2 | 10,90 | 5,10 | 2,10 | 0,90 | 0,22 |
| 3 | Athol | 31 | 7 | 1 | 2 | 2 | 7 | 1 | 21 | 0,90 | 8,10 | 2,10 | 19,90 | 0,19 |
| 4 | Clare A | 10 | 0 | 0 | 0 | 0 | 0 | 0 | 10 | 0,00 | 0,00 | 0,00 | 10,00 | ND |
| 5 | Clare B | 10 | 0 | 0 | 1 | 1 | 0 | 0 | 9 | 0,10 | 0,90 | 0,90 | 8,10 | 0,10 |
| 6 | Dixie | 10 | 1 | 3 | 1 | 1 | 1 | 3 | 5 | 0,80 | 1,20 | 3,20 | 4,80 | 1,00 |
| 7 | Seville B | 20 | 1 | 3 | 3 | 3 | 1 | 3 | 13 | 1,20 | 2,80 | 4,80 | 11,20 | 0,06 |
| 8 | Shorty | 10 | 2 | 1 | 0 | 0 | 2 | 1 | 7 | 0,20 | 1,80 | 0,80 | 7,20 | 1,00 |
| 9 | Thlavakisa | 10 | 0 | 0 | 0 | 0 | 0 | 0 | 10 | 0,00 | 0,00 | 0,00 | 10,00 | ND |
| 10 | Welverdiend A | 20 | 11 | 1 | 3 | 3 | 11 | 1 | 5 | 2,80 | 11,20 | 1,20 | 4,80 | 1,00 |
| 11 | Welverdiend B | 22 | 1 | 0 | 0 | 0 | 1 | 0 | 21 | 0,00 | 1,00 | 0,00 | 21,00 | ND |
| 12 | Seville A | 20 | 5 | 1 | 3 | 3 | 5 | 1 | 11 | 1,60 | 6,40 | 2,40 | 9,60 | 0,26 |
| 13 | Ludlow A | 20 | 4 | 0 | 0 | 0 | 4 | 0 | 16 | 0,00 | 4,00 | 0,00 | 16,00 | ND |
| 14 | Harrismith | 50 | 2 | 28 | 6 | 6 | 2 | 28 | 14 | 5,40 | 2,60 | 28,60 | 13,40 | 1,00 |
| 15 | Phuthaditjaba | 50 | 7 | 13 | 4 | 4 | 7 | 13 | 26 | 3,70 | 7,30 | 13,30 | 25,70 | 1,00 |
| 16 | Bergville | 50 | 16 | 3 | 15 | 15 | 16 | 3 | 16 | 11,20 | 19,80 | 6,80 | 12,20 | 0,03 |
| 17 | Manaleni | 20 | 1 | 6 | 6 | 6 | 1 | 6 | 7 | 4,20 | 2,80 | 7,80 | 5,20 | 0,16 |
| 18 | Boekenhouthoek | 17 | 2 | 0 | 4 | 4 | 2 | 0 | 11 | 1,40 | 4,60 | 2,60 | 8,40 | 0,01 |

**ND**, No statistics computed as the infection status was constant.
